# Systemische Sklerose: Eine Erkrankung mit vielen Gesichtern

**DOI:** 10.1007/s00105-025-05622-w

**Published:** 2025-12-11

**Authors:** Caroline Evers, Jörg Distler, Oliver Distler

**Affiliations:** 1 Klinik für Rheumatologie, Universitätsspital Zürich, Rämistr. 100, 8091 Zürich, Schweiz; 2 Klinik für Rheumatologie und Hiller-Forschungszentrum, Universitätsklinikum Düsseldorf, Moorenstr. 5, 40225 Düsseldorf, Deutschland

**Keywords:** Hautfibrose, Raynaud-Krankheit, Kollagenosen, Immunsuppressive Therapie, Antifibrotische Therapie, Skin fibrosis, Raynaud disease, Connective tissue diseases, Immunosuppression therapy, Antifibrotic therapy

## Abstract

Die systemische Sklerose (SSc) ist eine seltene rheumatologische Autoimmunerkrankung aus dem Kreis der Kollagenosen. Kennzeichnend ist eine Kombination aus Vaskulopathie und entzündlicher Fibrose von Haut und Organen. Die Erkrankung kann sich sehr heterogen präsentieren, was die Diagnosestellung erschwert und in der Überwachung und Therapie berücksichtigt werden muss. Dank Forschungsresultaten der letzten Jahrzehnte stehen mittlerweile deutlich mehr Medikamente für die Behandlung der SSc zur Verfügung. Die Wahl der Therapie ist bei jedem Erkrankungsfall an die individuelle Ausprägung anzupassen. Der vorliegende Beitrag bietet einen Überblick über die verschiedenen Krankheitsmanifestationen und Behandlungsmöglichkeiten bei SSc. Für eine bestmögliche Betreuung betroffener Patienten ist eine gute hausärztliche und rheumatologische Zusammenarbeit essenziell. Weitere internistische Fachärzte (Pneumologen, Gastroenterologen, Kardiologen, Nephrologen) müssen je nach Organbefall hinzugezogen werden.

## Abstract (en)

Systemic sclerosis (SSc) is a rare autoimmune connective tissue disease within the spectrum of rheumatologic disorders. It is characterized by a combination of vasculopathy and inflammatory fibrosis affecting the skin and various internal organs. The disease presents with heterogeneous manifestations, which can complicate diagnosis and must be carefully considered during both monitoring and treatment. In recent decades, advances in research have expanded the range of therapeutic options available for SSc. Treatment strategies need to be tailored individually, depending on the specific clinical manifestations in each patient. The aim of this article is to provide an overview of the diverse disease manifestations and current treatment approaches in SSc. For optimal patient care, close collaboration between rheumatologists, pneumologists, gastroenterologists, cardiologists, nephrologists and general practitioners is essential.

## Lernziele

Nach Lektüre dieses Beitrags

- sind Sie vertraut mit den möglichen Manifestationen der systemischen Sklerose (SSc).
- kennen Sie die Screeningalgorithmen für einzelne Organkomplikationen bei Patienten mit SSc.
- haben Sie einen Überblick über die verschiedenen Therapien und Medikamente, die bei SSc eingesetzt werden.
- kennen Sie die Risiken einer höher dosierten Glukokortikoidtherapie bei Patienten mit SSc.

## Einleitung

Die systemische Sklerose (SSc) ist mit einer Prävalenz in Europa von etwa 15/100.000 Einwohner eine **seltene Autoimmunerkrankung**

Seltene Autoimmunerkrankung

aus dem Kreis der **Kollagenosen**

Kollagenosen.

Pathogenetisch finden sich in Frühstadien der Erkrankung Endothelzelldysfunktionen, Autoantikörper und eine Dysregulation des Immunsystems, die schließlich zu einer vermehrten Produktion von Bindegewebe und Fibrose führen [1]. Möglich sind durchgehend milde Krankheitsausprägungen bis hin zu rasch progredienten, fulminanten Krankheitsverläufen mit Multiorganbefall, sodass eine rasche Diagnosestellung, Risikoabschätzung und gegebenenfalls Therapieeinleitung sowie ein regelmäßiges Monitoring von großer Relevanz sind.

### Infobox 1

Dies ist ein interdisziplinärer Beitrag, der die Sklerodermie aus rheumatologischer Sicht darstellt.

## Fallbeispiel

Eine 40-jährige Patientin stellt sich in Ihrer Hausarztpraxis mit seit einigen Monaten auftretenden kälteinduzierten Weiß-blau-Verfärbungen der Finger vor. Sie können während der Konsultation ein Raynaud-Phänomen beobachten und Ihnen fallen Hautverhärtungen an den Fingern und Teleangiektasien im Gesicht auf, sodass Sie eine Kollagenose vermuten. Die Laboruntersuchung ergibt erhöhte antinukleäre Antikörper (ANA) und positive Anti-Zentromer-Antikörper. Eine Kapillarmikroskopie ergibt eine Mikroangiopathie mit „frühem Sklerodermiemuster“. Sie veranlassen eine rheumatologische Beurteilung, in der eine limitiert-kutane SSc diagnostiziert wird. Ein Organscreening mit Elektrokardiogramm , Echokardiographie, Lungenfunktionsprüfung, Gehtest und Computertomographie (CT) des Thorax fällt unauffällig aus. Die Hautfibrose bleibt im Verlauf stabil, allerdings kommt es trotz Kälteschutzmaßnahmen sowie Medikation mit einem Kalziumantagonisten und im Verlauf mit einem Phosphodiesterase-5-Inhibitor wiederholt zu akralen Ulzera, die sich erst nach Addition eines Endothelinrezeptorblockers sowie durch monatliche Iloprost-Infusionen über die Wintermonate kontrollieren lassen.

Im Alter von 52 Jahren stellt sich die Patientin in Ihrer Praxis aufgrund einer schleichend zunehmenden Leistungsintoleranz mit Kurzatmigkeit unter Belastung vor. Herz- und Lungenauskultation sind unauffällig, die Blutuntersuchung bis auf ein leicht erhöhtes N‑terminales natriuretisches Propeptid vom B‑Typ (NT-proBNP) ebenfalls. Sie haben ein ungutes Gefühl und weisen die Patientin für eine vorgezogene Kontrolle zu. Im Organscreening bleibt die CT des Thorax unauffällig, die Lungenfunktion zeigt einen Abfall der Diffusionskapazität um 20 % und echokardiographisch bestehen Hinweise auf eine pulmonalarterielle Hypertonie, die schließlich mittels Rechtsherzkatheteruntersuchung bestätigt wird.

## Krankheitsmanifestationen

Die SSc ist eine **Multisystemerkrankung**

Multisystemerkrankung,

deren einzelne Manifestationen im Folgenden detaillierter ausgeführt sind. Typischerweise besteht eine Positivität für **antinukleäre Antikörper**

Antinukleäre Antikörper

(ANA). Verschiedene krankheitsspezifische Autoantikörpersubgruppen sind vorhanden und zeigen eine Häufung bei verschiedenen Krankheitsausprägungsformen: **Anti-Zentromer-Antikörper**

Anti-Zentromer-Antikörper

liegen häufiger bei limitiertem Hautbefall und assoziiert mit pulmonalarterieller Hypertonie vor, **Anti-Scl-70-Antikörper**

Anti-Scl-70-Antikörper

häufiger bei diffusem Hautbefall und assoziiert mit Lungenfibrose, während **Anti-RNA-Polymerase-III-Antikörper**

Anti-RNA-Polymerase-III-Antikörper

mit einem rasch progredienten Hautbefall, SSc-Nierenkrise und Malignomerkrankungen assoziiert sind [2, 3].

### Hautveränderungen

Eines der Hauptmerkmale der SSc ist die Hautfibrose, der die Erkrankung auch den ursprünglichen Namen „Sklerodermie“ zu verdanken hat. Basierend auf dem Ausmaß der Hautfibrose erfolgt eine Einteilung in die Unterformen **diffus-kutane SSc**

Diffus-kutane SSc

(dcSSc) mit Hautfibrose bis proximal der Ellbogen oder der Knie und **limitiert-kutane SSc**

Limitiert-kutane SSc

(lcSSc) mit Hautfibrose ausschließlich distal der Ellbogen bzw. Knie oder im Gesicht. Gelegentlich wird noch eine weitere Unterform abgegrenzt: die SSc sine scleroderma (ssSSc) ohne jegliche Hautfibrose; diese kann auch zur limitiert-kutanen Verlaufsform gezählt werden [4]. Zwar sind schwere Krankheitsverläufe mit bedrohlichem Organbefall auch bei Patienten ohne Hautfibrose möglich, dennoch können Ausmaß und Progredienz der Hautbeteiligung die generelle Krankheitsaktivität und das Ausmaß der Organbeteiligung widerspiegeln [5, 6].

Ein frühes Erkennungsmerkmal der Erkrankung sind oft **„puffy fingers“**

„Puffy fingers“,

die sich durch eine Schwellung der Finger noch ohne Hautsklerose auszeichnen. Auch bei Entwicklung von **Teleangiektasien**

Teleangiektasien

palmar, fazial und am Stamm sollte an eine SSc gedacht werden – insbesondere bei koexistentem Raynaud-Phänomen. Ebenfalls eine typische Hautmanifestation ist eine **Calcinosis cutis**

Calcinosis cutis

[7].

#### Merke

Bei einem Patienten mit SSc und rasch progredientem Hautbefall muss immer auch nach Zeichen eines progredienten Organbefalls gesucht werden, beispielsweise einer Lungenfibrose. Eine progrediente Lungenfibrose kann insbesondere in den Jahren nach einer Progredienz der Hautfibrose gehäuft auftreten.

### Kardiopulmonale Beteiligung

Etwa 50 % der Patienten mit SSc entwickeln im Krankheitsverlauf eine in der hochauflösenden Computertomographie (CT) erkennbare pulmonale Beteiligung im Sinne einer **interstitiellen Lungenparenchymerkrankung**

**Abb. 1:**
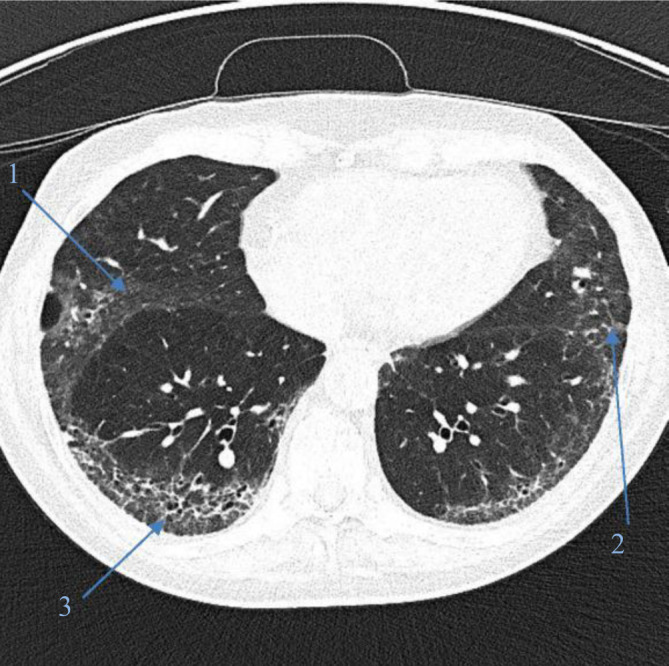
Lungenfibrose bei systemischer Sklerose mit Milchglastrübungen („Ground-glass“-Veränderungen) (*1*), Retikulationen (*2*) und Honigwabenmuster („honey combing“) (*3*)

Interstitielle Lungenparenchymerkrankung

(„interstitial lung disease“ [ILD]; Abb. 1); in Autopsien können auf histologischer Ebene bei bis zu 90 % der Patienten Lungengerüstveränderungen festgestellt werden [8]. Der Befall verläuft initial oft oligo- bis asymptomatisch, im Verlauf können Symptome wie Dyspnoe, chronischer Husten und Leistungsminderung/Fatigue auftreten. Regelmäßige Screeninguntersuchungen mit Lungenfunktionsprüfung, 6 min-Gehtest und CT-Schnittbildgebung der Lunge haben das Ziel, Einschränkungen und Fibrose möglichst früh zu erfassen. Die Diagnose wird auf Basis einer **hochauflösenden CT**

Hochauflösende Computertomographie

gestellt. Da die interstitiellen Veränderungen auf einem konventionellen Thoraxröntgenbild zumeist nicht oder zu spät sichtbar sind, wird dieses nicht mehr zum Screening auf ILD verwendet. Eine frühe Therapieeinleitung – teils vor Entwicklung von Symptomen – soll nach Möglichkeit eine Progression der Fibrose verhindern.

Eine weitere Lungenmanifestation ist die **pulmonale Hypertonie**

Pulmonale Hypertonie.

Diese tritt – im Gegensatz zur ILD – häufiger im späteren Krankheitsverlauf auf und bleibt initial ebenfalls klinisch inapparent. Gemäß Klassifikation [9] wird die pulmonale Hypertonie in fünf Klassen eingeteilt (Tab. 1). Bei der SSc tritt die **pulmonalarterielle Hypertonie**

Pulmonalarterielle Hypertonie

(PAH, Klasse I) bei etwa 10 % der Patienten auf, allerdings sind auch Klasse III (als Folge einer schweren ILD) oder Klasse II (im Rahmen einer Herzbeteiligung) möglich [10]. Für das Screening ist der Herzultraschall als alleinige Diagnostik nicht ausreichend, stattdessen werden zur Risikoabschätzung hinsichtlich des Vorliegens einer pulmonalen Hypertonie der multiparametrische **DETECT-Algorithmus**

DETECT-Algorithmus

[11] oder ähnliche Algorithmen verwendet. Die Diagnose wird mittels **Rechtsherzkatheteruntersuchung**

Rechtsherzkatheteruntersuchung

bestätigt.

**Tab. 1:** Einteilung der pulmonalen Hypertonie gemäß den Leitlinien der European Society of Cardiology (ESC) und European Respiratory Society (ERS) von 2022 [9]

Klasse I	Pulmonalarterielle Hypertonie (PAH)
Klasse II	Pulmonale Hypertonie assoziiert mit Linksherzerkrankungen
Klasse III	Pulmonale Hypertonie assoziiert mit Lungenerkrankungen/Hypoxie
Klasse IV	Pulmonale Hypertonie assoziiert mit pulmonalarterieller Okklusion
Klasse V	Pulmonale Hypertonie mit unklarer/multifaktorieller Genese

Eine schwere **kardiale Beteiligung**

Kardiale Beteiligung

im Sinne einer fulminanten entzündlichen Myokarditis oder höhergradige Herzrhythmusstörungen sind möglich. Auch eine diastolische Dysfunktion tritt bei Patienten mit SSc gehäuft auf [12].

#### Merke

Bei Patienten mit SSc und einer Verschlechterung der kardiopulmonalen Symptome oder klinischen Dekompensationszeichen muss neben einer möglichen Progression der Grunderkrankung auch an andere Ursachen gedacht werden, beispielsweise an einen respiratorischen Infekt unter Immunsuppression, eine konkomitante koronare Herzkrankheit oder eine medikamentöse Nebenwirkung wie Pneumonitis unter Methotrexattherapie.

### Vaskulopathie

Die Vaskulopathie ist ein Hauptmerkmal der SSc [13]. Praktisch alle Patienten mit SSc leiden an einem **Raynaud-Phänomen**

Raynaud-Phänomen.

Dies ist auch das mit Abstand häufigste Frühsymptom der Erkrankung. Mittels Kapillarmikroskopie lässt sich zumeist eine organische Mikroangiopathie mit typischem Sklerodermiemuster nachweisen. Etwa die Hälfte der Patienten entwickelt zudem **akrale Ulzera**

**Abb. 2:**
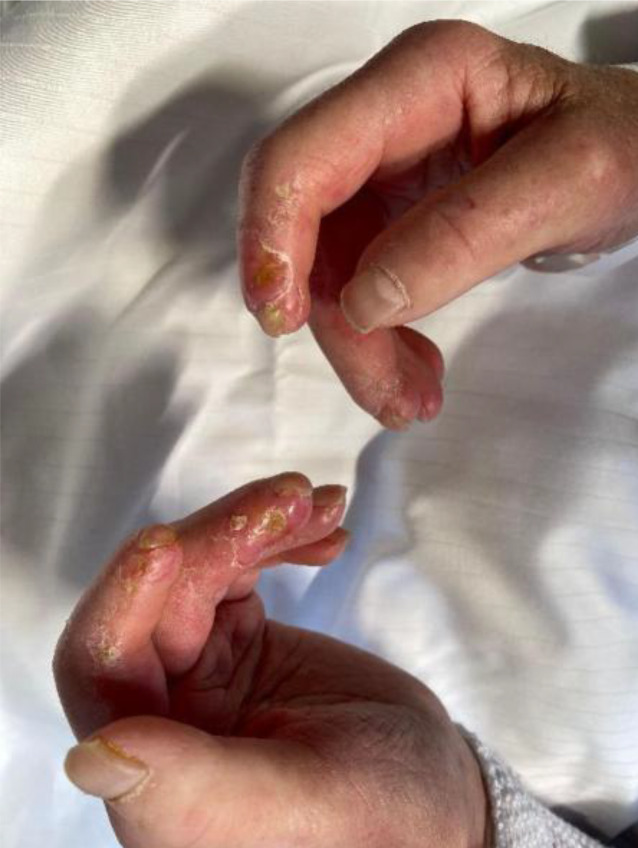
Multiple Handulzera bei systemischer Sklerose

Akrale Ulzera

(Abb. 2 und 3). Eine wichtige Komplikation der Vaskulopathie stellt die kritische Ischämie dar, die sich durch anhaltende Kälte, Zyanose und Ruheschmerzen im betroffenen Finger auszeichnet und einer raschen Behandlung bedarf.

**Abb. 3:**
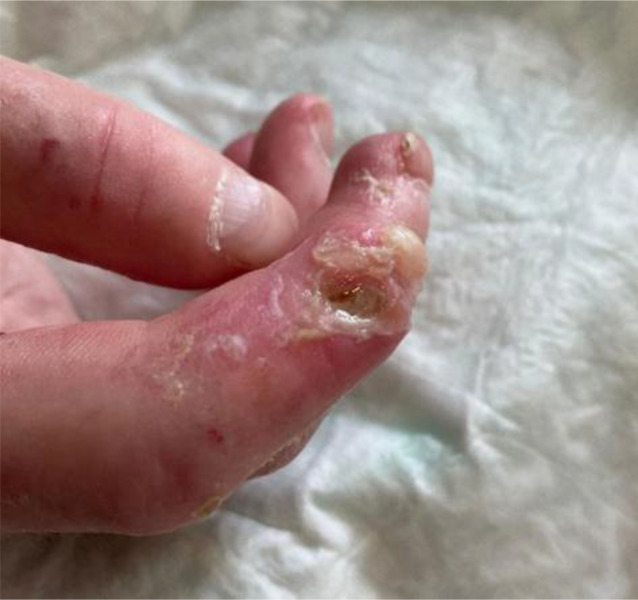
Großes und tiefes Ulkus am Zeigefinger bei systemischer Sklerose

#### Merke

Bei vermehrt schmerzhaften, purulenten Ulzera mit geröteter Wundumgebung und pulsierendem Schmerzcharakter muss an einen Wundinfekt gedacht werden und allenfalls eine Osteomyelitis mittels Magnetresonanztomographie ausgeschlossen werden. Bei der Behandlung von Wundinfektionen ist eine interdisziplinäre Behandlung von zentraler Bedeutung, da die Therapie von Infektion und Vaskulopathie im Rahmen der Grunderkrankung gemeinsamer Expertise bedarf.

### Gastrointestinale Beteiligung

Bei SSc sind gastrointestinale Symptome sehr häufig. Zugrunde liegt oft eine Motilitätsstörung, die zu Refluxbeschwerden, Dysphagie, verzögerter Magenentleerung, chronischer Obstipation sowie bakterieller Dünndarmüberwucherung („small intestinal bacterial overgrowth“ [SIBO]) mit Blähungen und wechselnder Stuhlkonsistenz sowie anorektaler Insuffizienz bis hin zu akuter/chronischer intestinaler Pseudoobstruktion führen kann [14].

#### Merke

Mittels oberer Endoskopie muss bei chronischem Reflux nach Barrett-Veränderungen des Ösophagus gesucht werden, bei Anämie nach einem Wassermelonenmagen („gastric antral vascular ectasia“ [GAVE]) oder intestinalen Angiodysplasien als möglichen Blutungsquellen [14].

### Renale Beteiligung

Eine Nierenbeteiligung im Sinne einer akuten **renalen Krise**

Renale Krise

bei SSc („scleroderma renal crisis“) tritt meist im frühen Krankheitsverlauf und häufiger bei Männern auf, insbesondere bei Patienten mit positiven RNA-Polymerase-III-Antikörpern. Eine höher dosierte Glukokortikoidtherapie (Prednison > 15 mg/Tag) ist ein wesentlicher Risikofaktor für die Entwicklung einer renalen Krise. Die renale Krise zeichnet sich in der Regel durch Nierenfunktionsverschlechterung, akuten Blutdruckanstieg (oft mit Endorganschäden) und hämolytische Anämie aus. Bleibende renale Schäden bis hin zur langfristigen Dialysepflichtigkeit bestehen bei mindestens der Hälfte der Patienten. Eine langsame **chronische Nierenfunktionsverschlechterung**

Chronische Nierenfunktionsverschlechterung

im Rahmen der Mikroangiopathie ist ebenfalls möglich [15].

#### Merke

Bei akutem Blutdruckanstieg bei bekannter SSc muss eine Nierenfunktionsverschlechterung gesucht werden. Bei entsprechendem Verdacht auf eine Nierenkrise ist eine sofortige Hospitalisation angezeigt.

### Weitere Krankheitsmanifestationen

**Muskuloskeletale Beteiligungen**

Muskuloskeletale Beteiligungen

bestehen primär in fibrosebedingten Gelenkkontrakturen, Arthralgien oder Arthritis, Tenosynovitiden und auch Myositis. Gehäuft bestehen **Sicca-Symptome**

Sicca-Symptome

der Augen und Mundschleimhäute, wobei die Beteiligung der Mundschleimhäute auch gehäuft zu Karies führt und zahnärztliche Behandlungen durch die verminderte Mundöffnung erschweren kann. Ebenfalls häufig sind **Allgemeinsymptome**

Allgemeinsymptome

wie Leistungsminderung und Fatigue.

#### Merke

Bei Gewichtsverlust muss an eine Tumorerkrankung gedacht werden, da gewisse Autoantikörperprofile bei SSc mit Malignomerkrankungen assoziiert sind, insbesondere Anti-RNA-Polymerase-III-Antikörper [2, 3].

## Behandlung der systemischen Sklerose

In Tab. 2 sind die Therapieempfehlungen der European Alliance of Associations for Rheumatology (EULAR) von 2023 schematisch dargestellt [16, 17]. Die einzelnen Behandlungen werden im Folgenden beschrieben.

**Tab. 2:** Vereinfachte schematische Darstellung der Therapieempfehlungen der European Alliance of Associations for Rheumatology (EULAR) 2023 bei systemischer Sklerose [16]

Evidenz	Raynaud-Phänomen	Digitale Ulzera	Pulmonalarterielle Hypertonie	Arthritis	Haut	Interstitielle Lungenerkrankung	Gastrointestinaltrakt	Niere
Hoch	PDE5i CCB Iloprost	PDE5i ERA Iloprost	PDE5i ERA Iloprost	–	RTX MTX	RTX MMF CYC Nintedanib	–	–
Mittel	–	–	Riociguat, Selexipag	–	MMF	TCZ	PPI	Keine ACEi zur Prävention
Gering	–	–	Keine Antikoagulation	–	TCZ	–	Prokinetika	ACEi
Expertenmeinung	–	–	–	MTX	–	–	Antibiotika	–

*ACEi* Angiotensin-converting-enzyme-Hemmer, *CCB* Kalziumkanalblocker, *CYC* Cyclophosphamid, *ERA* Endothelinrezeptorantagonist, *MMF* Mycophenolatmofetil, *MTX* Methotrexat, *PDE5i* Phosphodiesterase-5-Inhibitor, *PPI* Protonenpumpeninhibitor, *RTX* Rituximab, *TCZ* Tocilizumab

### Immunsuppressive und antifibrotische Therapie

Mittlerweile stehen verschiedene immunsuppressive Behandlungsmöglichkeiten zur Verfügung. Während **Methotrexat**

Methotrexat

primär bei Hautfibrose ohne begleitenden Organbefall oder bei Gelenkentzündungen eingesetzt wird, eignet sich **Mycophenolatmofetil**

Mycophenolatmofetil

zur Behandlung der Hautfibrose und der Lungenfibrose. Gemäß neueren Erkenntnissen eignet sich zudem eine Interleukin(IL)-6-Hemmer-Therapie mit **Tocilizumab**

Tocilizumab

insbesondere im frühen und entzündlichen Krankheitsstadium zur Behandlung einer rasch progredienten Lungenbeteiligung.

Therapeutische Effekte auf die Haut sind tendenziell vorhanden. **Rituximab**

Rituximab

kann ebenfalls bei Haut- und Lungenbeteiligung eingesetzt werden. Eine Therapie mit **Cyclophosphamid**

Cyclophosphamid

ist aufgrund eines etwas ungünstigeren Nebenwirkungsprofils im Vergleich zu Mycophenolatmofetil schweren Fällen mit bedrohlichem Organbefall (Lunge, Herz [18]) vorbehalten. In Fällen mit aggressivem Krankheitsverlauf, rasch entwickelter diffuser Hautfibrose und einer schlechten Prognose kann eine autologe hämatopoetische Stammzelltransplantation erwogen werden [16].

Bei Arthralgien oder Arthritis kann immunmodulierend **Hydroxychloroquin**

Hydroxychloroquin

eingesetzt werden, allerdings mit bislang geringer Evidenz [19]. Seit 2016/2017 besteht bei Lungenfibrose zudem die Möglichkeit einer Therapie mit **Nintedanib**

Nintedanib,

einem Tyrosinkinaseinhibitor, der antientzündliche und vor allem auch direkt antifibrotische Eigenschaften hat. Nintedanib kann additiv zur bestehenden Immunsuppression oder auch als Monotherapie bei Patienten mit Lungenfibrose eingesetzt werden [16]. Ein neuer Wirkstoff zur Therapie der ILD ist der Phosphodiesterase-4(PDE4B)-Inhibitor Nerandomilast, unter dem Patienten mit autoimmuner ILD Überlebensvorteile hatten. Die Zulassung dieses neuen Wirkstoffs ist abzuwarten [20].

Bei lebensbedrohlicher, therapieresistenter Lungenfibrose oder PAH mit schlechter Prognose ist in Einzelfällen auch eine **Lungentransplantation**

Lungentransplantation

zu erwägen, um das Gesamtüberleben zu verbessern [21].

#### Cave

Bei Patienten mit SSc sind Glukokortikoide (insbesondere in einer Dosis von Prednison > 15 mg/Tag) nach Möglichkeit zu vermeiden, da diese eine renale Krise triggern können [15].

#### Cave

Bei immunsupprimierten Patienten ist von hausärztlicher Seite eine regelmäßige Überprüfung und allenfalls Aktualisierung des Impfstatus wichtig, um einen bestmöglichen Infektionsschutz zu erreichen – wie auch die Klug-entscheiden-Empfehlung der Deutschen Gesellschaft für Innere Medizin von 2016 besagt [22]. Lebendimpfstoffe sind unter Immunsuppression kontraindiziert.

### Vasodilatative Therapie

In der Behandlung des Raynaud-Phänomens stehen an erster Stelle Kälteschutzmaßnahmen sowie das Vermeiden von vasokonstriktiven Medikamenten oder Nikotinkonsum. Medikamentös sind **Kalziumantagonisten**

Kalziumantagonisten

wie Nifedipin die erste Wahl. Alternativ – beispielsweise bei schlechter Verträglichkeit – ist der Einsatz des selektiven Serotoninwiederaufnahmehemmers **Fluoxetin**

Fluoxetin

[23] möglich, allerdings mit bislang niedriger Evidenz.

Bei Ulzera ist neben der Therapie des Raynaud-Phänomens auch eine lokale **Wundbehandlung**

Wundbehandlung

wichtig. Falls möglich ist eine Anbindung an eine Wundsprechstunde oder eine häusliche Behandlung durch einen Wundpflegedienst sinnvoll. Die Empfehlungen zur medikamentösen Therapie von Ulzera sind in Tab. 3 dargestellt. Therapeutisch werden **Phosphodiesterase-5-Inhibitoren**

Phosphodiesterase-5-Inhibitoren

wie Sildenafil (20–25 mg p.o. bis zu 3‑mal täglich) eingesetzt, welche die Ulzeraabheilung fördern und auch Raynaud-Beschwerden lindern. Der **Endothelinrezeptorantagonist**

Endothelinrezeptorantagonist

Bosentan (Dosierung initial 62,5 mg 2‑mal täglich, im Verlauf bei guter Verträglichkeit 125 mg 2‑mal täglich) wirkt prophylaktisch gegen die Entstehung neuer Ulzera. Eine **intravenöse Prostazyklintherapie**

Intravenöse Prostazyklintherapie

mit Iloprost (Dosierung 0,5–2 ng/kg pro min für 3–5 Tage) ist die Behandlung der Wahl bei schlecht heilenden Ulzera, zudem bei therapierefraktären Raynaud-Beschwerden und auch als Akuttherapie bei kritischer Ischämie. Des Weiteren gibt es unterstützende Daten für den Einsatz niedrig dosierter **Thrombozytenaggregationshemmer**

Thrombozytenaggregationshemmer

(Acetylsalicylsäure 100 mg täglich; [24]). Eine **therapeutische Antikoagulation**

Therapeutische Antikoagulation

mit unfraktioniertem Heparin kommt bei der Behandlung der kritischen Ischämie zum Einsatz [25].

**Tab. 3:** Medikation bei Fingerulzera gemäß Empfehlungen der European Alliance of Associations for Rheumatology (EULAR) von 2017/2023 [16].

Therapie von Ulzera (Abheilung)	Sekundärprophylaxe neuer Ulzera
Iloprost i.v.^a^	Bosentan^a^
Phosphodiesterase-5-Inhibitor^a^	Phosphodiesterase-5-Inhibitor
	Iloprost i.v.

^a^Entsprechend einer starken Empfehlung basierend auf qualitativ hochwertigen randomisierten, kontrollierten Studien und/oder Metaanalysen

Spezialtherapien mit bislang noch beschränkter Evidenzlage wie **lokale Botulinumtoxininjektionen**

Lokale Botulinumtoxininjektionen

[26] oder eine **digitale Sympathektomie**

Digitale Sympathektomie

[27] sind bei therapierefraktären Fällen zu erwägen. Bei pulmonalarterieller Hypertonie der Klasse I werden ebenfalls Phosphodiesterase-5-Inhibitoren wie Sildenafil oder Tadalafil sowie Endothelinrezeptorantagonisten wie Bosentan oder Macitentan eingesetzt. Eine **initiale Kombinationstherapie**

Initiale Kombinationstherapie

hat überzeugende Effekte auf das Überleben im Vergleich zu einer Einzeltherapie gezeigt und gilt heute als Therapie der Wahl. Schwere Fälle werden zusätzlich mit einer intravenösen Prostazyklintherapie behandelt.

Darüber hinaus stehen der oral verfügbare Prostazyklinrezeptoragonist Selexipag und der Stimulator der löslichen Guanylatzyklase **Riociguat**

Riociguat

zur Verfügung [16]. Ein neues Medikament zur Behandlung der PAH ist der Aktivininhibitor **Sotatercept**

Sotatercept,

der in mehreren randomisierten, kontrollierten Studien überzeugende Effekte auf zahlreiche PAH-Symptome und das Überleben gezeigt hat [28].

#### Merke

Akrale Ulzera erfordern oft eine Kombinationsbehandlung mit verschiedenen Medikamenten, begleitet von Kälteschutzmaßnahmen und lokaler Wundtherapie.

### Therapie der gastrointestinalen Beteiligung

Bei Refluxbeschwerden wird der Einsatz von **Protonenpumpeninhibitoren**

Protonenpumpeninhibitoren

(PPI) empfohlen. Bezüglich der Motilitätsstörungen können **Prokinetika**

Prokinetika

wie Metoclopramid oder Domperidon eingesetzt werden, allerdings mit mäßigem klinischem Effekt. Bei Hinweisen auf SIBO werden **rotierende Antibiotikatherapien**

Rotierende Antibiotikatherapien

in Zusammenarbeit mit einem spezialisierten Zentrum empfohlen [16], wobei die Datenlage bezüglich des Antibiotikaeinsatzes uneinheitlich ist.

#### Merke

Bei Patienten mit SSc und Refluxbeschwerden wird – nebst Lebensstilanpassungen – der niederschwellige Einsatz von PPI empfohlen, auch aufgrund wissenschaftlicher Hinweise, dass Reflux mit pulmonalen Mikroaspirationen zur rascheren Progression einer interstitiellen Lungenfibrose führen könnte [14].

### Therapie der renalen Krise

Bei der renalen Krise werden hoch dosiert **Angiotensin-converting-enzyme(ACE)-Hemmer**

Angiotensin-converting-enzyme-Hemmer

und falls notwendig andere Antihypertensiva eingesetzt. Die Behandlung sollte stationär erfolgen [15, 16].

#### Merke

Von einem prophylaktischen Einsatz von ACE-Hemmern bei Patienten mit SSc wird abgeraten, da Studien hier sogar einen schlechteren Verlauf und eine höhere Inzidenz der renalen Krise gezeigt haben [29].

### Weitere Therapien

Begleitend zu den oben aufgeführten medikamentösen Behandlungsoptionen sind auch **nichtmedikamentöse Therapiemaßnahmen**

Nichtmedikamentöse Therapiemaßnahmen

wichtig, wie regelmäßige **Physiotherapie**

Physiotherapie

zur Verbesserung und Bewahrung von Beweglichkeit und Muskelkraft, **Ergotherapie**

Ergotherapie

zwecks Verbesserung und Erhalt der Handfunktion, Gelenk- und Kälteschutzberatung sowie Entstauungsmaßnahmen bei „puffy fingers“. Gegebenenfalls wird eine **Ernährungstherapie**

Ernährungstherapie

zur Sicherstellung einer ausreichenden Nährstoffabdeckung durchgeführt, zudem möglicherweise auch **stationäre Rehabilitationsmaßnahmen**

Stationäre Rehabilitationsmaßnahmen.

Aufgrund der psychosozialen Belastung durch die Erkrankung kann auch eine ambulante **Psychotherapie**

Psychotherapie

oder die Anbindung an eine **Selbsthilfegruppe**

Selbsthilfegruppe

für Patienten mit SSc unterstützend wirken [30].

## Fazit für die Praxis

- Die systemische Sklerose (SSc) kann sich auf viele verschiedene Arten manifestieren. Hauptmerkmale der Erkrankung sind ein Raynaud-Phänomen sowie SSc-typische Hautveränderungen (Hautfibrose, „puffy fingers“, Teleangiektasien). Ein Organbefall ist ebenfalls häufig, insbesondere gastrointestinal und pulmonal.
- Eine rasche Progredienz des Hautbefalls kann mit dem aktiven Organbefall – parallel oder im weiteren Verlauf – korrelieren.
- Bei einem akuten Blutdruckanstieg muss an eine renale Krise gedacht und eine Nierenfunktionsverschlechterung abgeklärt werden.
- Es gibt ein breites Spektrum an immunmodulatorischen, primär antifibrotischen und symptomatischen Therapien.
- Glukokortikoide, insbesondere in Dosierungen von ≥ 15 mg täglich, sollen bei Patienten mit SSc vermieden werden, da sie ein Risikofaktor für das Auftreten einer renalen Krise sind.

## CME-Fragebogen

Bei einem Patienten mit erhöhten antinukleären Antikörpern (ANA) und Scl-70-Antikörper bestehen ein Raynaud-Phänomen und eine Hautfibrose der Hände bis zu den Oberarmen, Füßen und Unterschenkeln. Welche Diagnose stellen Sie basierend auf diesen Angaben?

Frühe systemische Sklerose

Systemische Sklerose sine scleroderma

Limitiert-kutane systemische Sklerose

Diffus-kutane systemische Sklerose

Undifferenzierte Kollagenose

Für welche Form der pulmonalen Hypertonie (gemäß ESC/ESR-Guidelines) stehen die meisten zugelassen Therapien zur Verfügung (*ESC* European Society of Cardiology, *ERS* European Respiratory Society)?

Klasse I

Klasse II

Klasse III

Klasse IV

Klasse V

An den Befall welches Organs muss bei einem akuten Blutdruckanstieg bei systemischer Sklerose gedacht werden?

Herz

Lunge

Lungengefäße

Niere

Nervensystem

Eine Patientin mit langjähriger, Anti-Zentromer-Antikörper-positiver systemischer Sklerose (SSc), schwerem Raynaud-Phänomen und Teleangiektasien, bislang ohne Herz‑/Lungenbeteiligung, klagt neu über eine progrediente Belastungsdyspnoe. Was ist die wahrscheinlichste SSc-assoziierte Ursache?

Pulmonalarterielle Hypertonie

Interstitielle Lungenerkrankung

Lungenembolie

Koronare Herzkrankheit

Linksherzinsuffizienz

Sie stellen in der Hausarztpraxis eine Anämie bei einem 42-jährigen Patienten mit limitiert-kutaner systemischer Sklerose fest. Reflux, Blut im Stuhl und B‑Symptome werden verneint. Was ist die wahrscheinlichste Ursache der Anämie?

Perniziöse Anämie

Barrett-Ösophagitis

Kolonkarzinom

Hämolyse

Eisenmangelanämie bei „gastric antral vascular ectasia“ (GAVE)

Welcher Autoantikörper ist mit einem erhöhten Risiko einer Malignomerkrankung assoziiert bei systemischer Sklerose?

Anti-Zentromer-Antikörper

Anti-Scl-70-Antikörper

Anti-RNA-Polymerase-III-Antikörper

Antinukleäre Antikörper

Rheumafaktor

Welches Medikament ist Ihre erste Wahl zur Behandlung des Raynaud-Phänomens?

Fluoxetin (selektiver Serotoninwiederaufnahmehemmer)

Sildenafil (Phosphodiesterase-5-Inhibitor)

Bosentan (Endothelinrezeptorantagonist)

Nifedipin (Kalziumantagonist)

Iloprost (Prostazyklinanalogon)

Welches Medikament wird gemäß den Empfehlungen der European Alliance of Associations for Rheumatology (EULAR) von 2023 bei SSc-Arthritis empfohlen (*SSc* systemische Sklerose)?

Mycophenolatmofetil

Methotrexat

Cyclophosphamid

Rituximab

Tocilizumab

Welches Medikament sollte insbesondere im frühen entzündlichen Krankheitsstadium zur Behandlung der interstitiellen Lungenerkrankung erwogen werden?

Nintedanib

Methotrexat

Mycophenolatmofetil

Tocilizumab

Cyclophosphamid

Wofür besteht erwiesenermaßen ein erhöhtes Risiko bei systemischer Sklerose im Falle einer Therapie mit Prednison 20 mg täglich?

Renale Krise

Intestinale Pseudoobstruktion

Progression der „interstitial lung disease“

Verschlimmerung einer pulmonalarteriellen Hypertonie

Zunahme der Hautfibrose
